# Two-Stage Surgical Management for Acutely Presented Large Vestibular Schwannomas: Report of Two Cases

**DOI:** 10.3390/brainsci13111548

**Published:** 2023-11-04

**Authors:** Abdullah Keles, Burak Ozaydin, Ufuk Erginoglu, Mustafa K. Baskaya

**Affiliations:** Department of Neurological Surgery, University of Wisconsin—Madison School of Medicine and Public Health, Madison, WI 53792, USA; abdullah.keles@wisc.edu (A.K.); burakozaydin@icloud.com (B.O.); erginoglu@wisc.edu (U.E.)

**Keywords:** facial nerve, nervus intermedius, retrosigmoid approach, staged surgery, translabyrinthine approach, vestibular schwannoma

## Abstract

The surgical management of vestibular schwannomas should be based on their presentation, neuro-imaging findings, surgeons’ expertise, and logistics. Multi-stage surgery can be beneficial for large-sized lesions with acute presentations. Herein, we highlighted the indications for two cases managed initially through the retrosigmoid and, subsequently, translabyrinthine approaches. The first case presented with acute balance and gait issues and a long history of hearing loss and blurred vision. Neuroimaging findings revealed a cerebellopontine angle lesion, resembling a vestibular schwannoma, with significant brainstem compression and hydrocephalus. Due to the rapidly deteriorating clinical status and large-sized tumor, we first proceeded with urgent decompression via a retrosigmoid approach, followed by gross total resection via a translabyrinthine approach two weeks later. The second case presented with gradually worsening dizziness and hemifacial numbness accompanied by acute onset severe headaches and hearing loss. Neuroimaging findings showed a large cerebellopontine angle lesion suggestive of a vestibular schwannoma with acute intratumoral hemorrhage. Given the acute clinical deterioration and large size of the tumor, we performed urgent decompression with a retrosigmoid approach followed by gross total resection through a translabyrinthine approach a week later. Post-surgery, both patients showed excellent recovery. When managing acutely presented large-sized vestibular schwannomas, immediate surgical decompression is vital to avoid permanent neurological deficits.

## 1. Introduction

Vestibular schwannomas (VSs) account for 90–95% of tumors found in the cerebellopontine angle (CPA). Depending on their size and location, these tumors present with a range of symptoms. These can be as mild as mild headaches, tinnitus, vertigo, disequilibrium, and mild unilateral hearing loss, whereas large VSs might present with severe symptoms of other cranial nerves (mainly trigeminal, facial, and lower cranial nerves), intentional tremor, gait ataxia, and hydrocephalus. Furthermore, sizeable tumors can occasionally bleed internally, resulting in sudden onset hearing loss, new or exacerbated symptoms of cranial nerves, CPA syndrome, and even sudden alterations in mental status [1].

The treatment of VSs should be individualized for every patient. Depending on the patient’s specifics, observation, surgery, radiosurgery, or a combination of these can be chosen. Elderly asymptomatic patients with tumors confined to the internal auditory canal (IAC) are best suited for observation. Regardless of tumor size, surgery aiming for complete tumor removal offers a cure while preserving the facial nerve functionality. For those who have residual tumors or with small- to medium-size tumors who cannot tolerate surgery, radiosurgery is a viable option.

For the surgical management of VSs, three main approaches can be utilized: the retrosigmoid, the translabyrinthine, and the middle fossa approach, or sometimes combinations or variations of these [1,2,3,4,5]. Besides these most widely used approaches, there are other combinations and variations that could be utilized in some selected cases, but with limited indications [6,7,8,9]. Factors such as the size and extension of the tumor and the patient’s hearing status play crucial roles in the choice of surgical approach.

Regardless of the selected surgical approach, surgical treatment of large VSs (>3 cm) presents significant challenges. Surgical treatment of large VSs is often associated with higher morbidity, increased complications, and less favorable facial nerve outcomes compared to smaller VSs [10,11,12]. For the surgical treatment of large VSs, two-stage surgery was introduced to enhance the outcomes, and several two-stage surgical series have been published [10,11,12,13,14,15,16].

In this report, we present two large VS cases in which both initially underwent emergency surgery using the retrosigmoid approach, followed by an elective secondary procedure for gross total removal through the translabyrinthine approach. However, the rationale for the staging varied between the two cases. We highlight these cases to illustrate the criteria for employing multi-stage surgeries to manage large VSs.

## 2. Results

### 2.1. Case 1

A 52-year-old man presented with a progressive decline in his left ear hearing and blurry vision over the past 18 months. In addition, he recently began to experience balance and gait problems which had notably worsened in the past few weeks. Following an episode of dizziness that resulted in a fall and subsequent emergency room admission, a CT scan of the head revealed a large lesion in the left CPA. His initial audiogram showed sensorineural hearing loss in the left ear, accompanied by a total lack of word recognition.

**Figure 1:**
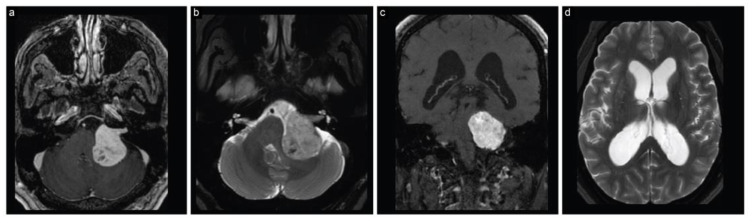
Case 1, preoperative neuroimaging. (**a**) Axial T1-weighted postcontrast, (**b**) axial T2-weighted, (**c**) coronal T1-weighted postcontrast, and (**d**) axial T2-weighted MRI scans show a large CPA lesion suggesting a vestibular schwannoma along with hydrocephalus.

The patient immediately had a cranial MRI that displayed a sizeable (4 cm × 3.5 cm × 3.3 cm) left CPA lesion, consistent with a vestibular schwannoma (Figure 1). The lesion caused marked compression on the brainstem and fourth ventricle, resulting in hydrocephalus.

Considering the large size of the tumor, the significant compression it caused on the brainstem and fourth ventricle, the accompanying hydrocephalus, and the rapidly deteriorating balance and gait problems, we decided to urgently commence with initial decompression through a retrosigmoid approach. We had also planned for an elective second stage to achieve gross total resection using a translabyrinthine approach (Supplementary Video).

Before the initial surgery, a ventriculostomy catheter was inserted in the left frontal region.

With the patient positioned supine, we slightly rotated the head to the right and used neuronavigation to mark the locations of the sigmoid and transverse sinuses. We then made a postauricular C-shape skin incision and lifted a myocutaneous flap. Subsequently, a retrosigmoid craniotomy was carried out using a single burr hole, located just below the junction of the sigmoid and transverse sinus.

The dura was opened in a C-shaped fashion, followed by the draining of cerebrospinal fluid from the lateral cerebellomedullary cistern to relax the cerebellum. As the arachnoid dissection progressed, the tumor came into view. Once we confirmed the facial nerve-free tumor regions with a facial nerve stimulator, the tumor capsule was incised. Central tumor debulking commenced with the use of an ultrasonic aspirator and piecemeal resection using microsurgical techniques. As we proceeded with alternating debulking and dissection, cranial nerves IX and X, the basilar artery, and the origin of the anterior inferior cerebellar artery (AICA) were visualized and carefully detached from the tumor. After sufficient tumor debulking, the pressure on the neurovascular structures was relieved, hemostasis was achieved, and the wound was closed in standard fashion.

**Figure 2:**
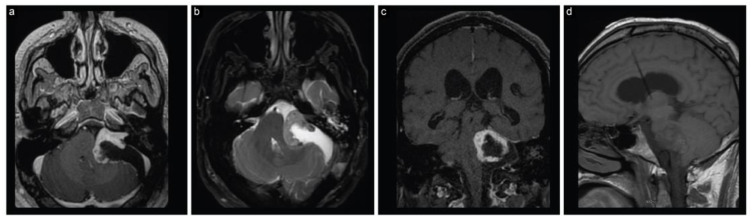
Case 1 post-stage 1 neuroimaging findings. (**a**) Axial T1-weighted postcontrast, (**b**) axial T2-weighted, (**c**) coronal T1-weighted postcontrast, and (**d**) sagittal T1-weighted MRI scans show partial resection with decompression of the brainstem and surrounding structures along with regressed hydrocephalus.

The patient woke up with House–Brackmann grade I facial nerve function. The histopathological diagnosis was confirmed as a WHO grade I schwannoma. An early postoperative MRI revealed partial resection of the tumor and decompression of the brainstem and surrounding structures, along with regressed hydrocephalus (Figure 2).

Two weeks afterward, we initiated the second stage of the surgery, the translabyrinthine approach, to achieve gross total resection. The patient was positioned supine, and the previous wound was reopened. Bony exposure, labyrinthectomy, and 270-degree IAC drilling were performed. Afterward, the IAC dura was incised and the intracanalicular part of the tumor was exposed. By employing facial nerve stimulation, the intracanalicular part of the facial nerve was mapped out and carefully detached from the tumor. We then proceeded with presigmoid dural opening, which revealed the tumor and prior resection cavity. At the upper boundary of the tumor, the trigeminal nerve was identified and detached from the tumor. At the lower boundary of the tumor, the vestibulocochlear nerve and the lower cranial nerves were discerned and separated from the tumor. We then delineated the tumor–brainstem border, and the facial nerve root exit zone was detected and confirmed with a nerve simulator. Further piecemeal tumor resection allowed us to find the brainstem origin of the vestibulocochlear nerve in the pontomedullary sulcus.

Once the facial nerve was entirely separated from the tumor, the rest of the tumor was removed en bloc, and hemostasis was achieved. Following that, we removed the incus and packed the eustachian tube with previously harvested stripes of temporalis fascia to avoid postoperative cerebrospinal fluid (CSF) leak. The post-resection cavity was then packed using abdominal fat grafts that had been previously harvested.

As part of our standard neuromonitoring protocol, we continuously monitor and record the compound muscle action potential (CMAP) of the orbicularis oculi and orbicularis oris muscles. In addition, throughout surgery, we use a facial nerve stimulator with a minimum stimulation threshold (MST) of 0.05–0.1 mA for the identification and verification of the facial nerve, for assessing the proximity to the nerve, for monitoring the nerve integrity, and for predicting the postoperative facial nerve outcome. In the majority of the VS cases, the facial nerve could be detached from the tumor by employing gentle traction-counter traction, along with microvascular sharp dissection techniques.

**Figure 3:**
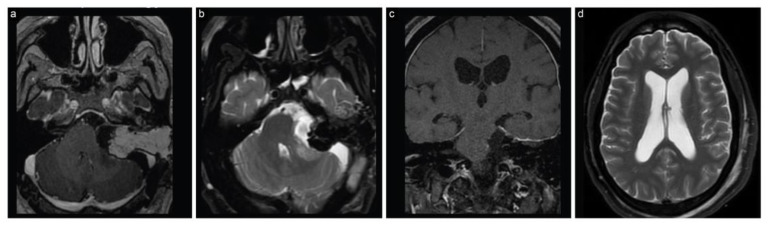
Case 1 post-stage 2 neuroimaging findings. (**a**) Axial T1-weighted postcontrast, (**b**) axial T2 fat suppressed, (**c**) coronal T1-weighted, and (**d**) axial T2-weighted MRI scans show gross total resection with decompression of the brainstem and surrounding structures along with decreased hydrocephalus.

The patient woke up with House–Brackmann grade 2 facial nerve function without any new neurological deficits. The patient’s postoperative recovery was uneventful and he eventually had normal facial nerve function at the 3-month follow-up. A postoperative MRI confirmed a gross total resection (Figure 3).

### 2.2. Case 2

**Figure 4:**
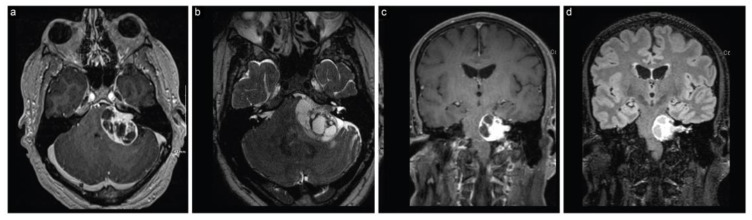
Case 2 preoperative neuroimaging. (**a**) Axial T1-weighted postcontrast, (**b**) axial T2-weighted, (**c**) coronal T1-weighted postcontrast, and (**d**) coronal T2 FLAIR MRI scans show a large cystic CPA lesion, suggesting a vestibular schwannoma along with recent intratumoral hemorrhage.

The second case is a 48-year-old man who had presented with dizziness and numbness on the left side of his face for the past six weeks. These symptoms had been escalating, and were accompanied by a severe, newly developed headache. An MRI scan revealed a large cystic left cerebellopontine angle lesion (4.1 cm × 2.5 cm × 2.8 cm), suggestive of a VS, along with signs of recent hemorrhage within the lesion (Figure 4). An initial audiogram revealed moderate to severe sensorineural hearing loss, with no serviceable hearing on the left side. 

Given the recent intratumoral hemorrhage and significant tumor size, which resulted in acute increased compression of the surrounding neurovascular structures, we decided to proceed with a two-stage approach. Initially, we performed emergency decompression via the retrosigmoid approach, followed by an elective translabyrinthine approach for a gross total resection (Supplementary Video). Once the patient was positioned supine and the head was slightly rotated, a standard C-shape skin incision, myofascial flap elevation, and craniotomy for a retrosigmoid approach were performed. The tense posterior fossa dura was cautiously incised. The cerebellum was notably swollen because of the heightened pressure in the posterior fossa caused by the tumor and recent intratumoral hemorrhage. We then navigated the lateral cerebellomedullary cistern and drained the CSF to relax the posterior fossa, which facilitated the exposure of the tumor.

Once we verified the area was clear of the facial nerve using nerve stimulation, we incised the tumor capsule. We then accessed the hemorrhagic cavity and removed its content. After sufficient tumor debulking, hemostasis was achieved, and the surgical wound was closed in layers.

**Figure 5:**
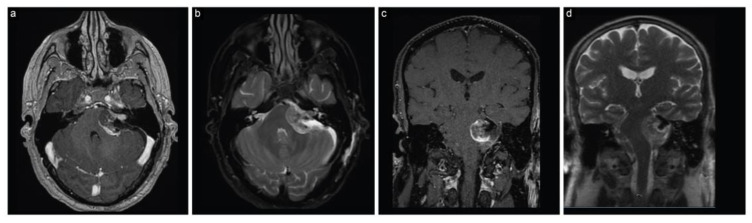
Case 2 post-stage 1 neuroimaging findings. (**a**) Axial T1-weighted postcontrast, (**b**) axial T2-weighted, (**c**) coronal T1-weighted postcontrast, and (**d**) coronal T2-weighted MRI scans show partial resection with decompression of the brainstem and surrounding structures, with evacuation of the cystic and hemorrhagic portion of the lesion.

The patient woke up with House–Brackmann grade I facial nerve function. Histopathological analysis identified it as a WHO grade I schwannoma. An early postoperative MRI displayed a decompressed brainstem and extent of resection (Figure 5).

A week afterward, we initiated the second planned stage to achieve gross total removal through a translabyrinthine approach. The second stage began with the placement of a lumbar drain and obtaining a fat graft from the abdomen. As detailed in the previous case, opening of the previous wound, bony exposure, labyrinthectomy, 270-degree IAC drilling, and intracanalicular tumor removal were performed. We then proceeded with an incision of the presigmoid dura. Once the presigmoid dura opened, the tumor came into view. Then, extending the arachnoid dissection around the tumor, we exposed and released the trigeminal nerve in the upper part of the tumor.

Upon confirming the facial nerve-free zone, the tumor capsule was incised, and piecemeal removal was commenced. Notably, the remaining tumor was less vascular than in the initial stage. On the lateral part of the surgical field, cranial nerves VII and VIII became visible. The facial nerve was identified with nerve stimulation and the vestibulocochlear nerve was cut. As we proceeded with the tumor removal, the nervus intermedius came into view. Every attempt should be made to preserve the nervus intermedius when it is encountered during surgery.

We then turned our attention to the IAC portion of the tumor. We traced the facial nerve in the IAC and meticulously detached it from the tumor, employing sharp and semi-sharp microsurgical dissection techniques. Subsequently, all the remaining arachnoid adhesions were cleared, and the rest of the tumor was removed en bloc. At the final stage of surgery, the facial nerve was stimulated at 0.05 milliampere in the brainstem zone as well as along its entire length. We then proceeded with wound closure. 

**Figure 6:**
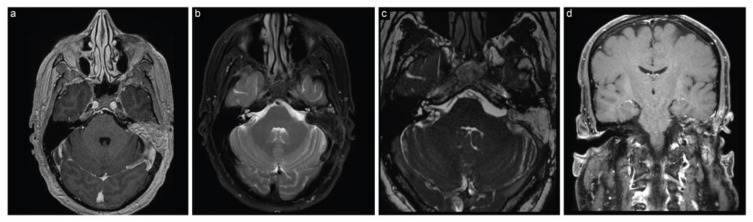
Case 2 post-stage 2 neuroimaging findings. (**a**) Axial T1-weighted postcontrast, (**b**) axial T2-weighted, (**c**) axial FIESTA, and (**d**) coronal T1-weighted postcontrast MRI scans show gross total resection with decompression of the brainstem and surrounding structures.

Postoperative examinations revealed House–Brackmann grade 1 facial nerve function and an early postoperative MRI confirmed the gross total resection of the tumor (Figure 6). The patient remained tumor-free with normal facial nerve function at the 1-year follow-up.

## 3. Discussion

The first successful complete removal of a VS was carried out by Balance in 1894 and reported in 1907 [14,17]. Later, in 1913, pioneer neurosurgeons, Horsley of London, v. Eiselsberg of Vienna and Krause of Berlin presented their VS surgical outcomes at the International Congress of Medicine in London. They reported mortality rates of 67%, 77% and 84%, respectively. Given the challenging nature of the cases, surgical interventions were commonly performed in two stages, notably by v. Eiselsberg and Horsley [14].

In 1917, Cushing introduced his revolutionary technique of partial removal through the intracapsular enucleation method, which remarkably reduced the mortality rates first to 35%, and later further to 11% [18]. In 1925, Dandy shared his expertise on 23 VSs, which he had performed since 1915. He incorporated the finger technique to achieve gross complete removal after the intracapsular enucleation [14]. With the advent of the microsurgery era, new microsurgical approaches were introduced by House (translabyrinthine approach), Kurze and Doyle (subtemporal transpetrosal approach), and Rand and Kurze (suboccipital transmeatal approach) [19,20,21]. Since the early days of microsurgery, numerous case series have been published in the literature [4,22,23,24,25,26,27,28,29,30,31,32].

The introduction of intraoperative electromyography for monitoring the facial nerve, along with new techniques, has not entirely resolved the complexities of preserving facial nerve functionality, especially in cases with large tumors. Even with a success rate of 86–92% in preserving the anatomical continuity of the facial nerve, there are instances where patients experience facial nerve disfunctions after surgery, even when their nerves are anatomically preserved [10,28,33,34,35,36]. Tumors larger than 3 cm often have less favorable outcomes, while those 2 cm or less have better outcomes [10,25,33]. Samii et al. reported a 100% gross total removal rate, with a 44% rate of good facial nerve outcome in cases larger than 3.9 cm in size [28]. In addition, a recent study by Gazia and colleagues revealed an association between tumor size, MST, CMAP, and facial function in the short and long term, which aids in improving predictions of facial performance [37].

When dealing with large and complex VSs, it is essential to consider staged surgeries preoperatively. Besides the tumor size, various other factors can complicate the surgical approaches to VSs. These include variations in vascular and bony anatomy (thrombosed or missing sigmoid sinus, elevated jugular bulb, anteriorly positioned sigmoid sinus, and a contracted or small mastoid bone), existence of cystic components, and brainstem compression, along with hydrocephalus, intratumoral hemorrhage, prior treatments (either surgical or stereotactic radiation), the tumor’s vascularity, and the patient’s pre-existing comorbidities [1,12,13].

In the literature, studies have shown that for selective large VSs, staged surgeries improve facial nerve outcomes, reduce morbidity, and have a higher likelihood of achieving gross total removal [10,11,12,13,15,16]. One potential rationale for achieving better outcomes with staged surgeries might be that the most delicate dissections can be performed separately, minimizing the impact of surgeon fatigue. Often, detaching the tumor from the facial nerve in the IAC and brainstem, the most important part of the surgery, takes place towards the end of the surgery. Although multi-stage surgery may not be scheduled in advance, it should be considered intraoperatively if a surgeon experiences fatigue during the most crucial parts of the surgery. This might be the safest and most efficient solution for the selected cases.

In prior published series, two-stage surgeries were predominantly performed for the treatment of large vestibular schwannomas. Given that these patients exhibited severe hearing loss preoperatively, hearing preservation was not the primary goal in these surgeries. In these same series, a lower or no additional incidence of morbidity and mortality was associated with staged surgery for the resection of large vestibular schwannomas [10,12,15]. Abe et al. reported a postoperative increase in hearing loss for both of their presented cases where the tumors originated from vestibular nerves [13].

Tinnitus is one of the most common initial symptoms of VSs along with hearing loss, occurring in 60–80% of VS patients [1]. In some of those patients, tinnitus could worsen and significantly influence a patient’s quality of life [38,39,40,41]. In the published two-stage surgeries for vestibular schwannomas series, no postoperative tinnitus was reported, as seen in our cases.

During the surgical resection of large tumors, it is not always possible to determine the origin of the tumor. As illustrated in our video, sometimes even the identification of the vestibular nerve branches and cochlear nerve is not feasible. Therefore, during our surgeries, we cut the vestibulocochlear nerve to achieve gross total resection. In cases where the cochlear nerve can be preserved, cochlear implantation should be considered in the same sittings [42,43,44,45].

### Indications and Timing Interval for the Staged Surgeries

As of now, there remains a lack of consensus on the indications and the timing between the two stages in the management of VSs [10,15].

Raslan et al. made staging decisions intraoperatively based on facial nerve anatomical condition and stimulation, tumor adherence, as well as the conditions of the brainstem and cerebellum. They determined the time interval based on facial nerve outcome after the first stage. For those with a House–Brackmann grade I and II, the second stage was scheduled between two and four weeks after the first stage, while the remainder waited for six months, aiming to maximize their recovery in the interim [15]. In contrast, Comey et al. chose to perform second stage surgery once postoperative imaging studies verified the residual tumor’s decompression away from the pons. Their time interval between the stages ranged widely from 0.5 to 32 weeks, averaging 4.5 weeks [10]. On the other hand, Abe et al. recommended determining the timing of the second stage based on verifying decreases in vascularity for cases with hypervascularity [13].

Ideally, the second stage surgery should be conducted prior to the formation of arachnoid adhesions or tumor revascularization [31]. To prevent such adhesions, a thin gelatin sponge layer can be used during the initial surgery’s closure [46]. In our experience, the second stage should not be delayed more than 2–3 weeks at maximum to avoid arachnoid adhesion. As documented in various studies, the remaining tumor often exhibits decreased vascularity and less adherence, which allows for a relatively easier dissection in the second stage surgery [10,13,14]. This was observed in our second case as well.

In theory, multi-stage surgeries could bring additional operative risks related to multiple anesthesia administration, potential surgical site infections, and issues with wound recovery. However, in both the published series and our series, there were no evident complications due to the aforementioned reasons.

Lastly, rapidly gathering neuro-otology and neurosurgery teams might not be viable in some emergency cases. In such scenarios, a multi-stage approach must be strongly considered to minimize the risk of permanent neurological deficits and ensure the best patient care.

In summary, the literature suggests that a two-stage surgical approach for large VSs offers numerous advantages. In selected cases, this enhances facial nerve outcomes, reduces patient morbidity, and increases the likelihood of complete tumor removal. With a two-stage approach, surgeons can tackle intricate dissections with more focus, thereby minimizing mistakes from extended operations. Moreover, in urgent scenarios, like those we have experienced where rapid setup and team assembly were challenging, the two-stage approach ensures that patients receive the best care without compromising the intricacy of the procedures or the necessary preparations.

## 4. Conclusions

In summary, even in cases with large VSs, a single-stage gross total removal is feasible if the tumor has well-defined arachnoid borders and appropriate consistency. Immediate surgical decompression should be performed for selected large and acutely presented VSs to achieve gross total removal with little or no additional morbidity.

## Data Availability

Not Applicable.

## Supplementary Materials

The following supporting information can be downloaded at: https://www.mdpi.com/article/10.3390/brainsci13111548/s1, Video S1: Two-stage Surgical Management for Acutely Presented Large Vestibular Schwannomas: Report of Two Cases with Operative Video Demonstrations.
