# Supplementary material for: Two-Stage Surgical Management for Acutely Presented Large Vestibular Schwannomas: Report of Two Cases
Source: Brain Sci. 2023 Nov 4;13(11):1548. doi: 10.3390/brainsci13111548 (PMC10669422; doi:10.3390/brainsci13111548)
Supplement: Supplementary file 1 [file brainsci-13-01548-s001.zip › brainsci-2658254-supplementary.pdf]

Supplementary material is a video

https://doi.org/10.5281/zenodo.8378328
